# Investigation of potential targets of *Porphyromonas* CRISPRs among the genomes of *Porphyromonas* species

**DOI:** 10.1371/journal.pone.0183752

**Published:** 2017-08-24

**Authors:** Takayasu Watanabe, Masaki Shibasaki, Fumito Maruyama, Tsutomu Sekizaki, Ichiro Nakagawa

**Affiliations:** 1 Laboratory of Food-borne Pathogenic Microbiology, Research Center for Food Safety, Graduate School of Agricultural and Life Sciences, The University of Tokyo, 1-1-1 Yayoi, Bunkyo-ku, Tokyo, Japan; 2 Department of Oral Implantology and Regenerative Dental Medicine, Graduate School of Medical and Dental Sciences, Tokyo Medical and Dental University, 1-5-45 Yushima, Bunkyo-ku, Tokyo, Japan; 3 Department of Microbiology, Graduate School of Medicine, Kyoto University, Yoshida-Konoe-cho, Sakyo-ku, Kyoto, Japan; Oregon Health & Science University, UNITED STATES

## Abstract

The oral bacterial species *Porphyromonas gingivalis*, a periodontal pathogen, has plastic genomes that may be driven by homologous recombination with exogenous deoxyribonucleic acid (DNA) that is incorporated by natural transformation and conjugation. However, bacteriophages and plasmids, both of which are main resources of exogenous DNA, do not exist in the known *P*. *gingivalis* genomes. This could be associated with an adaptive immunity system conferred by clustered regularly interspaced short palindromic repeat (CRISPR) and CRISPR-associated (*cas*) genes in *P*. *gingivalis* as well as innate immune systems such as a restriction-modification system. In a previous study, few immune targets were predicted for *P*. *gingivalis* CRISPR/Cas. In this paper, we analyzed 51 *P*. *gingivalis* genomes, which were newly sequenced, and publicly available genomes of 13 *P*. *gingivalis* and 46 other *Porphyromonas* species. We detected 6 CRISPR/Cas types (classified by sequence similarity of repeat) in *P*. *gingivalis* and 12 other types in the remaining species. The *Porphyromonas* CRISPR spacers with potential targets in the genus *Porphyromonas* were approximately 23 times more abundant than those with potential targets in other genus taxa (1,720/6,896 spacers vs. 74/6,896 spacers). *Porphyromonas* CRISPR/Cas may be involved in genome plasticity by exhibiting selective interference against intra- and interspecies nucleic acids.

## Introduction

Homologous recombination is a major event for gaining new genes into bacterial genomes and for altering genome structure [1–3]. *Helicobacter pylori* and *Neisseria meningitidis* are well-known pathogenic bacteria with panmictic population structures [4–7], and recombination events between the two random genomes are predominant [8]. These species are characterized by flexibility and plasticity in their genomes, which occurs by altering gene content and genome structure rather than by conserving them clonally [3, 9]. Conjugative transfer introduces DNA into bacterial cells [10–12], whereas natural competence is also important for introducing extracellular DNA [13, 14].

However, the CRISPR/Cas system is a bacterial adaptive immune system against exogenous nucleic acids such as bacteriophages and plasmids [15]. This system defends its own genomes [3], as well as innate immune systems in bacteria. The restriction-modification system is one of them, and cleaves exogenous DNA by recognizing specific nucleotide sequences [16]. Recent studies for the CRISPR/Cas system have revealed various functions of Cas proteins encoded by *cas* genes adjacent to CRISPR arrays [17, 18]. Cas homology and *cas* gene arrangements are used to classify CRISPR/Cas. In addition, several studies suggest noncanonical functions of CRISPR/Cas such as transcriptional regulation [19, 20] or the regulation of biofilm formation [21].

The oral bacterial species *Porphyromonas gingivalis* is a pathogen of periodontal inflammatory diseases and requires a strict anaerobic condition to grow [22]. *Porphyromonas gingivalis* may be panmictic rather than clonal because this species possesses a diverse gene composition and genome structure among strains [23, 24]. Natural transformation and conjugative transfer are the proposed routes of introducing exogenous DNA into *P*. *gingivalis* cells [25–27], and phage transduction is generally considered the route in bacteria [28]. By contrast, there are no known prophages and plasmids for *P*. *gingivalis*, based on its genomic information [29, 30]. This factor could be because the immune systems such as CRISPR/Cas and restriction-modification system in *P*. *gingivalis* genomes [31–33] may protect against the intracellular persistence of these exogenous elements [34]. However, our previous study for identifying and analyzing *P*. *gingivalis* CRISPRs demonstrated many CRISPR spacers for which potential targets were not determined, and that a limited number of these spacers had potential targets in *P*. *gingivalis* genomes [35]. In this study, we determined CRISPR/Cas in *P*. *gingivalis* and other *Porphyromonas* genomes. We then identified potential targets of CRISPR spacers in a public nucleotide database and in these *Porphyromonas* genomes. The *Porphyromonas* genomes provided a large number of potential targets.

## Materials and methods

### Bacterial strains, culture conditions, and the extraction of their genomic DNA

We used 51 *P*. *gingivalis* isolates, which were used for genetic typing and intraspecies diversity analyses in our previous study (S1 Table) [35]. Culture conditions of these isolates and the extraction method of their genomic DNA have previously been described [35].

### Determination of the draft genome sequences

The extracted genomic DNA of 51 *P*. *gingivalis* isolates was processed with the TruSeq DNA Sample Prep Kit (Illumina, San Diego, CA, U.S.A) or with the Nextera DNA Library Preparation Kit (Illumina) for high-throughput sequencing on the Illumina Genome Analyzer IIx (101-bp paired-end reads) or MiSeq platforms (250-bp paired-end reads), respectively. Before sequencing, the libraries were quantified by real-time polymerase chain reaction on the LightCycler (Roche Diagnostics, Indianapolis, IN, U.S.A.) with the LightCycler FastStart DNA Master SYBR Green I (Roche Diagnostics) and KAPA Library Quantification kits for Illumina (Kapa Biosystems, Wilmington, MA, U.S.A.), and were qualitatively verified by capillary electrophoresis on the Bioanalyzer (Agilent Technologies, Santa Clara, CA, U.S.A.) with the High Sensitivity DNA Kit (Agilent Technologies). The obtained sequence reads were deposited at the DDBJ/EMBL/GenBank under the accession number DRX019659-DRX019709. The reads were checked for quality using FastQC (http://www.bioinformatics.babraham.ac.uk/projects/fastqc/) and were trimmed and filtered using Trimmomatic v0.22 [36] using the following parameters: ILLUMINACLIP:2:20:10, LEADING:10, TRAILING:10, SLIDINGWINDOW:4:15, and MINLEN:50. Among the trimmed/filtered reads, the overlapping paired-end read pairs were joined using fastq-join v1.1.2–537 [37] with default parameters. The trimmed/filtered reads were then assembled into contigs using Velvet (v1.2.10) [38] with a minimum contig length of 400. The *k*-mers of the odd values were tested for assembling each genome, as follows: 31–99 for data from the Genome Analyzer IIx and 151–249 for data from the MiSeq. Among *k*-mers with the top five longest N50, the *k*-mer with the least number of contigs was finally used for assembly (S1 Table). The obtained contigs were annotated using the RAST server v2.0 [39, 40] with the following options: FIGfam release70, automatically fix errors, fix frameshifts, build the metabolic model, and backfill gaps.

### Prediction of CRISPR arrays and CRISPR-associated (*cas*) genes

For predicting CRISPR arrays, we used the following data: the aforementioned 51 *P*. *gingivalis* draft genome sequences and 59 complete/draft genome sequences of *Porphyromonas* species obtained from the National Center for Biotechnology Information (NCBI) GenBank database. The 59 genomes consisted of 13 genomes of *P*. *gingivalis* and 46 genomes of 14 known and 10 unclassified *Porphyromonas* species (S1 Table). In addition to the genomes determined in this study, genomes downloaded from the NCBI GenBank were annotated using the RAST server v2.0. The CRISPR arrays were extracted from the data using the CRISPR Recognition Tool (v1.2) [41] with a minimum repeat length of 30, maximum repeat length of 80, and maximum spacer length of 80. To enhance the prediction accuracy as much as possible, we set these parameters with the following criteria: (1) the exclusion of any repeats in non-CRISPR regions and (2) the inclusion of CRISPR repeats and spacers longer than the default parameter 38 bp of maximum repeat length and maximum spacer length. The predicted CRISPR arrays were further subjected to a nucleotide 6-frame translation Basic Local Alignment Search Tool (BLASTX) search against the NCBI GenBank Non-redundant Protein Database to exclude repeat motifs in protein-coding sequences with the threshold of e-value ≤1e-50. The *cas* genes were predicted using a protein BLAST (BLASTP) search of protein-coding sequences (CDSs) against the NCBI Non-redundant Protein Database, and were verified with the NCBI Conserved Domain Database, as described previously [35]. Genetic organizations of the CRISPR arrays and the identified *cas* genes were visualized by *in silico* Molecular Cloning Genomics Edition (IMC-GE) v4.3.1 (In Silico Biology, Kanagawa, Japan) [42]. In the visualized organizations, *cas* genes were colored in accordance with colors used in a previous report [17].

### Determination of the repeat types among the predicted CRISPR arrays

The program CD-HIT-EST (v4.6) [43, 44] was used for clustering all predicted CRISPR repeats and for determining the consensus repeat sequence of each cluster, based on the threshold of sequence identity 0.8. The obtained clusters were verified manually. For classifying each repeat cluster and predicting repeat orientation, an integrated web server (i.e., CRISPRmap v2.1.3–2014 and CRISPRstrand) was used [45, 46]. Each repeat cluster was named with the length value of the consensus repeat sequence and the serial number within the length value as the repeat type. For instance, four clusters with the consensus repeat sequences of 30 bp were named as types 30.1, 30.2, 30.3, and 30.4. The presence of nucleotide sequences in any bacterial genomes significantly similar to the CRISPR repeats was searched using nucleotide BLAST (BLASTN) of the consensus repeats against the NCBI Nucleotide Collection. Nucleotide polymorphism in each repeat type was examined using WebLogo v3 in which entropy of each nucleotide character was calculated as a bit value [47].

### Prediction of the CRISPR targets in a public nucleotide database and in the *Porphyromonas* genomes used in this study

We subjected all CRISPR spacers predicted in this study to a BLASTN search against bacterial/archaeal/viral nucleotide sequences in the NCBI Nucleotide Collection with the parameters of word size 7 and dust filter off. Instead of using a bit score to evaluate the search results, as described previously [35], we considered query coverage and nucleotide mismatches by using the following criteria. In the search results, the sequences with 100% query coverage were considered potential CRISPR targets, and nucleotide mismatch was counted for each alignment of the query and potential target. In considering the detection of multiple potential targets from a single spacer, the number of potential targets was counted, as well as that of the spacer itself. The number of CRISPR spacers with potential targets was visualized with the number of nucleotide mismatch by bar charts. These methods for predicting CRISPR targets were also used for the search against the *Porphyromonas* genomes used in this study.

Among spacers with potential targets in the *Porphyromonas* genomes, we examined the relationship between each spacer and its targets. We distinguished spacers with targets in the same species (i.e., “intraspecies targets”) from spacers with the targets in different species (i.e., “interspecies targets”). Spacers with intraspecies targets were further categorized as follows: (1) targets were “endogenous” when the spacer and its targets were on the same genome and (2) targets were “exogenous” when they were on different genomes. The number of spacers with intraspecies or interspecies targets and with endogenous or exogenous targets was counted, and their relationships were visualized on a Venn diagram. The relationships between the CRISPR-harboring and target-harboring species and the endogenicity were visualized by a heat map. In each repeat type, nucleotide polymorphism upstream and downstream 50-bp of the potential targets was examined using WebLogo v3 to predict protospacer-adjacent motifs (PAMs). In the WebLogo examination, we excluded repeat types with no upstream or downstream sequence or a single upstream or downstream sequence of the potential targets that resulted from nucleotide ambiguity or insufficient nucleotide length in the draft genomes.

## Results

### Predicted CRISPR arrays among *Porphyromonas gingivalis* and 24 *Porphyromonas* species

We determined the draft genome sequence of 51 isolates to predict unknown potential CRISPR arrays. The values of GC content and the number of CDS in the 51 draft genomes were in agreement with those in 13 complete/draft publicly available genomes of *P*. *gingivalis* (S1 Table). The CRISPR arrays were predicted in these 64 *P*. *gingivalis* genomes and in 46 other genomes in 22 *Porphyromonas* species, which included 14 known and eight unclassified species (S2 and S3 Tables). Possible truncation or interruption of CRISPR arrays in draft genomes could result in the overcounting of CRISPR arrays, although 435 CRISPR arrays and 7,331 spacers were detected from *Porphyromonas* genomes (S4 Table).

We next clustered all CRISPR repeats detected in this study by nucleotide sequence similarity for grouping of CRISPR arrays. Using CD-HIT-EST v4.6, the 7,331 repeats in the 435 CRISPR arrays were clustered into 18 repeat types (S5 Table). The consensus repeat sequences of the 18 types varied from 30 to 47 bp (S5 Table). The number of spacers per CRISPR locus varied among species and among repeat types (S1 Fig, S3 Table). The repeats in type 30.1 were the most abundant among all repeats, which would result from the abundance of type 30.1 repeats in *P*. *gingivalis*, the genomes of which were most abundantly included in this study. Among the 18 repeat types, types 30.1, 36.1, 36.2, and 37.1 corresponded with previously described types 30, 36.1, 36.2, and 37, respectively, on *P*. *gingivalis* genomes [35] (S5 Table). The consensus nucleotide sequence existed in each of the 18 repeat types, although several nucleotide loci were highly heterogenous in several types such as 30.4, 32.1, and 47.1 (S2 Fig). In the CRISPRmap v2.1.3–2014, all 18 consensus repeats, except for type 30.2, were classified with any of the CRISPRmap IDs, structural motifs, sequence families, and superclasses (S5 Table). The consensus repeat of type 30.2 was not classified in the CRISPRmap, although the CRISPR array of type 30.2 did not have an amino acid sequence similarity against any proteins in the NCBI Non-redundant Protein Database, which indicated that the array would not be any repetitive motifs of proteins. The 18 consensus repeats did not exhibit significant nucleotide sequence similarity with any bacterial/archaeal genomes other than *Porphyromonas* species in the NCBI Nucleotide Collection, except for type 32.2. The consensus repeat of type 32.2 exhibited 100% nucleotide sequence similarity of the entire 32 bp with *Parabacteroides distasonis* strain ATCC 8503 genome and 100% similarity of sequential 31 bp with *Paenibacillus stellifer* strain DSM 14472 genome. In these two non-*Porphyromonas* genomes, the regions significantly similar with type 32.2 consensus repeat were clustered as 5 in *Parabacteroides distasonis* and 23 in *Paenibacillus stellifer*, and were interspaced by approximately 30 bp or 40 bp, which suggest that these regions were the repeats in CRISPR arrays in the two genomes.

### Prediction of *cas* genes among *Porphyromonas gingivalis* and 24 *Porphyromonas* species

**Fig 1:**
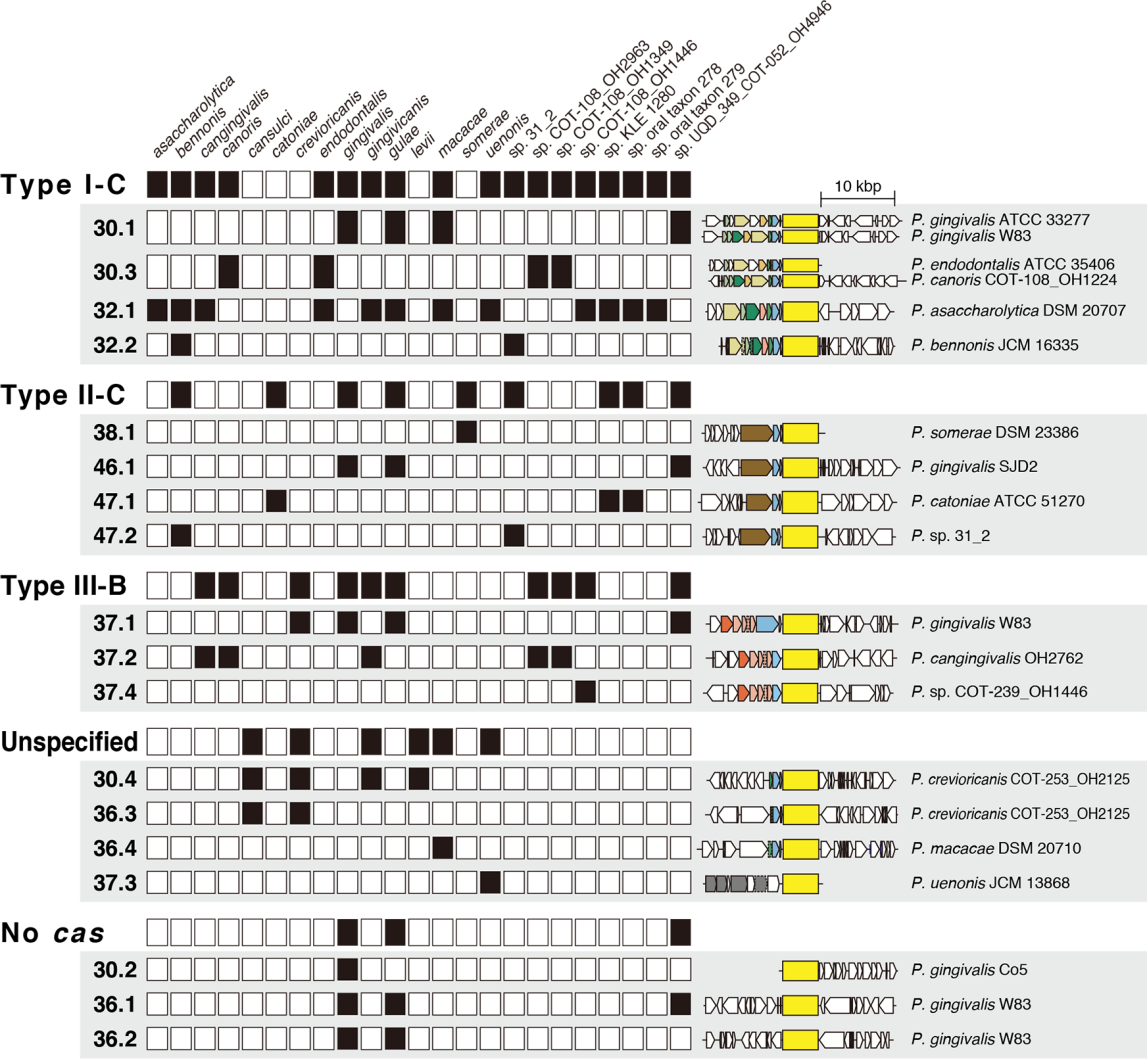
Eighteen repeat types of CRISPR/Cas detected in this study and their distribution among *Porphyromonas* species with their corresponding *cas* types. In each (CRISPR-associated) *cas* type, the presence or absence of each repeat type is shown for each *Porphyromonas* species by a black/white box. The boxes on the right side of the *cas* type name show the presence or absence of each *cas* type, regardless of repeat type. Representative genetic organization of CRISPR arrays and *cas* genes is shown for each repeat type with the name of *Porphyromonas* species. Two genetic organizations are shown if two representatives are hard to regard as the same organization in a particular type. The CRISPR arrays are indicated by yellow boxes. The CDSs are indicated by arrows, and CDSs of the predicted *cas* genes are colored, as described in the Materials and Methods section. The broken arrows indicate *cas* genes that are untypable using our criteria. All genetic elements are proportional to their nucleotide lengths, except for the CRISPR arrays.

We examined the presence and location of *cas* genes on the CRISPR-harboring *Porphyromonas* genomes. The *cas* genes were found adjacent to CRISPR arrays in 15 of 18 repeat types (Fig 1). Using the classifications of a previous report [17], we classified the *cas* gene arrays of 11 repeat types, whereas the *cas* classifications were unspecifiable in four repeat types (Fig 1, S5 Table). The *cas* presence and classifications of the four repeat types 30.1, 36.1, 36.2, and 37.1 were consistent with the previous report of the *P*. *gingivalis cas* genes; the *cas* genes were absent in types 36.1 and 36.2, and the *cas* classifications of types 30.1 and 37.1 were I-C and III-B, respectively [35]. In each of the 15 repeat types, most *cas* gene arrays showed synteny among the genomes, except for types 30.1 and 30.3 (S3 Fig). The gene arrangement was heterogenous to exhibit two independent representatives in each of these two types, although the *cas* classifications were the same for the two representatives as I-C.

Among the *cas* classifications from I to III, those observed in this study were limited to the three classifications of I-C, II-C, and III-B (Fig 1, S5 Table). An interesting relation was observed between the *cas* classifications and the nucleotide lengths of the consensus repeats (i.e., most repeat types with the consensus repeat lengths of ≤32 bp, 37 bp, and ≥38 bp were classified as I-C, III-B, and II-C, respectively; S5 Table). In this manner, the information from the CRISPRmap showed such relations to the nucleotide length of the repeats (S5 Table). When observing the *cas* classifications in each *Porphyromonas* species, there was no specificity in the *cas* classifications to any specific *Porphyromonas* species (Fig 1). Most species possessed several *cas* classifications such as *P*. *bennonis* (e.g., I-C and II-C) and *P*. *gingivicanis* (e.g., I-C and III-B) (Fig 1). Among the species, all three classifications of I-C, II-C, and III-B were found in *P*. *gingivalis*, *P*. *gulae*, and *Porphyromonas* sp. UQD_349_COT-052_OH4946 (Fig 1).

### Potential CRISPR targets in the public nucleotide database and *Porphyromonas* genomes

**Fig 2:**
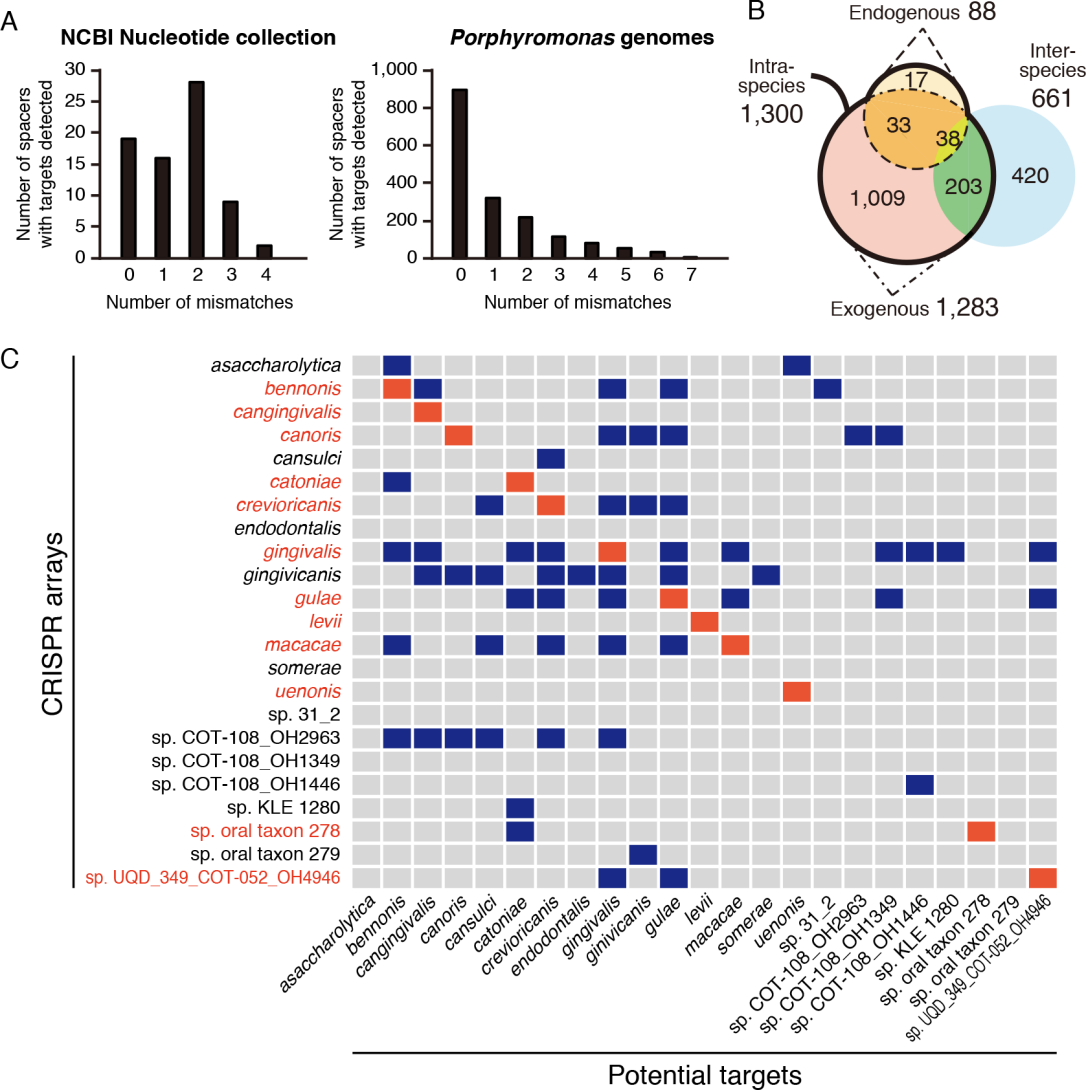
The number of potential targets in the NCBI Nucleotide Collection and in the *Porphyromonas* genomes, and dissection of the latter group by focusing on the locational relationships between the CRISPR spacer and its potential targets. (A) The number of potential targets of the *Porphyromonas* CRISPR arrays in the NCBI Nucleotide Collection and in the *Porphyromonas* genomes are shown in the left and right bar charts, respectively. In each chart, the numbers are presented, based on the number of mismatches. The numbers for the *Porphyromonas* genomes are excluded from the chart for the NCBI Nucleotide Collection. (B) The Venn diagram shows the relationships among spacers having intraspecies or interspecies targets and endogenous or exogenous targets. Two circles for spacers with endogenous and exogenous targets are overlapped on the left side to form one large population of spacers with intraspecies targets. This circle is overlapped with one circle on the right for spacers with interspecies targets. For instance, the number 33 indicates spacers with both endogenous and exogenous targets but without any interspecies targets. (C) The relationships between CRISPR spacers and their potential targets in *Porphyromonas* species. The left side presents the name of species with CRISPR arrays, and the lower axis shows species with potential targets of the CRISPR spacers in the genomes of the species on the left. The presence or absence of potential targets is indicated by blue or gray, respectively, and those that are targeted by CRISPR spacers of the same species are indicated by red.

We searched potential CRISPR targets in bacterial/archaeal/viral nucleotide sequences in the NCBI Nucleotide Collection. As expected from the previous report in which few CRISPR targets were predicted [35], the potential targets in genomes other than the genus *Porphyromonas* existed for only 1.1% of the spacers (74/6,896) (S6 Table). The targets were in 53 known genera and one unclassified genus, the former of which included 52 bacterial and seven archaeal species with a wide variety of habitats such as human oral cavity, human intestinal tract, animals, and natural environment (Table 1). The number of nucleotide mismatches between the spacer and its targets ranged from 0 to 4, and the number of spacers decreased with an increase in the number of mismatches, although the number of spacers was the largest when the number of mismatches was 2 (Fig 2A).

**Table 1:** Microbial species whose genomic regions were targeted by *Porphyromonas* CRISPR spacers.

Species of spacer	Species of CRISPR target
***Porphyromonas asaccharolytica***	*Methylobacterium extorquens*, *Niabella soli*, *Synechococcus phage*
***Porphyromonas bennonis***	*Bacteroides fragilis*
***Porphyromonas cangingivalis***	*Borrelia bissettii*, *Chitinophaga pinensis*, *Ferroplasma acidarmanus*, *Listeria monocytogenes*
***Porphyromonas canoris***	*Myxococcus fulvu*s, *Sphingobacterium* sp.
***Porphyromonas endodontalis***	*Thermotoga* sp.
***Porphyromonas gingivalis***	*Acetobacter pasteurianus*, *Acholeplasma oculi*, *Bacteroides salanitronis*, *Belliella baltica*, *Brevibacillus laterosporus*, *Campylobacter jejuni* subsp. *jejuni*, Candidatus *Liberibacter americanus*, *Capnocytophaga canimorsus*, *Clostridium botulinum*, *Clostridium cellulovorans*, *Clostridium saccharoperbutylacetonicum*, *Clostridium thermocellum*, *Desulfurella acetivorans*, *Ensifer adhaerens*, *Flavobacterium psychrophilum*, *Helicobacter hepaticus*, *Leptospira interrogans*, *Methanobrevibacter ruminantium*, *Polaromonas* sp., *Providencia stuartii*, *Rahnella* sp., *Sanguibacter keddieii*, Uncultured bacterium clone LM0ACA20ZH11FM1, *Winogradskyella* sp., *Xenorhabdus nematophila*
***Porphyromonas gingivicanis***	*Paenibacillus odorifer*
***Porphyromonas gulae***	*Borrelia parkeri*, *Burkholderia cepacia*, Candidatus *Nitrosopumilus*, *Desulfosporosinus acidiphilus*, *Lactobacillus buchneri*, *Sorangium cellulosum*, *Streptococcus mitis*, *Thermococcus* sp., *Thermofilum* sp., Uncultured *Desulfobacterium*, Uncultured *Flavobacteriia*, *Winogradskyella* sp.
***Porphyromonas levii***	*Bacteroides salanitronis*, *Pseudomonas fluorescens*
***Porphyromonas macacae***	*Bacillus halodurans*, *Campylobacter coli*, *Carboxydothermus hydrogenoformans*, *Elizabethkingia anophelis*, *Sulfolobus solfataricus*
***Porphyromonas uenonis***	*Halorhabdus tiamatea*, *Streptomyces lividans*
***Porphyromonas* sp. 31_2**	*Bacteroides fragilis*, *Parabacteroides distasonis*
***Porphyromonas* sp. COT_108-OH2963**	*Francisella guangzhouensis*
***Porphyromonas* sp. UQD_349_COT-052_OH4946**	*Arcobacter nitrofigilis*, *Enterococcus faecalis*

In our previous study, the potential targets of CRISPR spacers of *Porphyromonas gingivalis* were primarily in their own genomes [35]. To further characterize the CRISPR arrays with the potential targets, we searched the targets among the 110 *Porphyromonas* genomes used in this study. The potential targets for 24.9% of the spacers (1,720/6,896) were in the genomes of 21 *Porphyromonas* species (S7 Table). We could not find any potential targets for the remaining spacers ([74.0%] 5,102/6,896). The number of spacers, as well as the spacers and their potential targets in the NCBI Nucleotide Collection, decreased with an increase in the number of mismatches up to 7 (Fig 2A, S8 Table). Nucleotide polymorphisms did not show any obvious patterns suggesting PAMs (S4 Fig).

Two types of targeting styles existed in spacers with potential targets in the *Porphyromonas* genomes: 75.6% of the spacers (1,300/1,720) were characterized by the intraspecies targets, and 38.4% (661/1,720) were characterized by interspecies targets (Fig 2B). The former group was further divided into 88 spacers with endogenous targets and 1,283 spacers with exogenous targets (Fig 2B). These numbers included overlaps of spacers across intra- and interspecies targets or across endogenous and exogenous targets (Fig 2B). The spacers and their potential targets existed among various *Porphyromonas* species (Fig 2C). The number of spacers with endogenous targets was nearly 14.5 times lower than that of exogenous targets (88:1,283); however, spacers with the endogenous targets existed in 12 of 23 *Porphyromonas* species (Fig 2C).

We further examined whether the potential targets in the *Porphyromonas* genomes were in the CDSs or intergenic regions. Nearly one-half ([57.0%] 980/1,720) of the spacers had potential targets in CDSs encoding hypothetical proteins, whereas 26.3% (453/1,720) of the spacers had targets in the CDSs with a known function (Table 2). The remaining spacers ([16.7%] 287/1,720) had potential targets in the intergenic regions (Table 2). As mentioned previously, three-quarters ([75.6%] 1,300/1,720) of the spacers had intraspecies targets, and the number of spacers with endogenous targets was quite small ([5.1%] 88/1,720) (Table 2). The CDSs with a known function that harbored the CRISPR targets included 20 CDSs, which could be associated with exogenous elements such as bacteriophages and conjugative transposons, and were primarily characterized as intraspecies targets (Table 2). These CDS encoding phage-related proteins were annotated because of homology with the proteins in bacteriophages with hosts other than *Porphyromonas* species. However, most of these 20 CDSs are adjacent to those encoding conjugative transposon-related proteins, and the remaining in 20 CDSs are separate from the conjugative transposon-related CDSs and are unlikely to be intact prophages or conjugative transposons [31–33].

**Table 2:** Function of *Porphyromonas* genomic regions where CRISPR targets are located.

Function	Number of spacer		
	With the intra-species targets	With the endogenous targets	Total
**3-hydroxybutyryl-CoA dehydratase**	1	1	1
**3-oxoacyl-[acyl-carrier-protein] synthase, KASIII**	1	1	1
**5-Enolpyruvylshikimate-3-phosphate synthase**	-	-	1
**Acetyl-CoA synthetase (ADP-forming) alpha and beta chains, putative**	2	2	2
**Adenine-specific methyltransferase**	7	-	10
**Aldehyde dehydrogenase**	4	-	4
**Alkanesulfonates ABC transporter ATP-binding protein / Sulfonate ABC transporter, ATP-binding subunit SsuB**	3	3	3
**ATPase involved in DNA repair**	1	1	1
**ATPase involved in DNA repair, phage associated**	3	-	3
**Carboxynorspermidine dehydrogenase**	4	4	4
**ClpB protein**	3	3	3
**Conjugative transposon protein TraG**	-	-	1
**Cysteine desulfurase, SufS subfamily**	1	-	1
**DNA double-strand break repair Rad50 ATPase**	3	-	5
**DNA methylase N-4/N-6 domain protein**	1	1	1
**DNA polymerase I**	-	-	1
**DNA polymerase III alpha subunit**	2	2	2
**DNA primase**	47	-	55
**DNA-cytosine methyltransferase**	2	-	3
**Ferric siderophore transport system, periplasmic binding protein TonB**	2	2	2
**Glutamate formiminotransferase @ Glutamate formyltransferase**	1	1	1
**Glycerophosphoryl diester phosphodiesterase, phage variant**	5	-	5
**Hydrolase, putative**	4	-	4
**Immunoreactive 43 kDa antigen PG32**	1	1	1
**Integrase**	7	-	11
**Integrase/recombinase**	18	-	19
**Integrase/recombinase (XerC/CodV family)**	1	-	1
**Large Subunit Ribosomal RNA; lsuRNA; LSU rRNA**	1	1	1
**Leucyl-tRNA synthetase**	1	1	1
**Long-chain-fatty-acid—CoA ligase**	1	-	1
**Magnesium and cobalt efflux protein CorC**	-	-	1
**Metallo-beta-lactamase superfamily domain protein in prophage**	-	-	1
**Mobile element protein**	3	-	3
**Myosin heavy chain**	-	-	1
**N-acetylmuramoyl alanine amidase**	13	-	19
**N-acetylmuramoyl-L-alanine amidase**	4	-	4
**NAD(P) transhydrogenase subunit beta**	-	-	1
**Oxidoreductase, Gfo/Idh/MocA family**	1	1	1
**Peptidase S49**	-	-	1
**Phage (Mu-like) virion morphogenesis protein**	9	-	12
**Phage antirepressor protein**	-	-	2
**Phage portal protein**	3	-	6
**Phage protein**	25	1	63
**Phage tail length tape-measure protein**	47	2	63
**Phage terminase, large subunit**	3	-	3
**Phage terminase, large subunit @ intein-containing**	15	-	15
**Phage-related protein**	24	1	25
**Phosphomannomutase / Phosphoglucosamine mutase**	1	1	1
**Phosphoribosylformylglycinamidine synthase, synthetase subunit / Phosphoribosylformylglycinamidine synthase, glutamine amidotransferase subunit**	1	1	1
**Portal protein, phage associated**	3	-	6
**Predicted thiamin transporter PnuT**	2	2	2
**Probable peptidase**	-	-	1
**Prophage Lp2 protein 6**	-	-	2
**Protein gp49, replication initiation [Bacteriophage A118]**	-	-	4
**Protein of unknown function DUF114**	5	2	5
**Putative antirepressor protein**	1	-	1
**Putative ATP-dependent helicase**	-	-	3
**Putative carboxy-terminal processing protease**	1	1	1
**Putative integrase**	1	-	2
**Putative methyltransferase**	3	-	5
**Putative phage repressor**	-	-	3
**Putative terminase large subunit**	3	-	4
**Putative tetratricopeptide repeat family protein**	1	-	1
**RecA protein**	1	1	1
**Ribonuclease HI**	-	-	3
**Ribonucleotide reductase of class Ia (aerobic), alpha subunit**	1	1	1
**Secretion activator protein, putative**	-	-	1
**Signal peptide peptidase SppA, 36K type**	1	-	1
**Site-specific DNA-methyltransferase**	6	-	13
**SohB protein, peptidase U7 family**	1	-	1
**SusC, outer membrane protein involved in starch binding**	-	-	1
**Thioredoxin family protein**	1	1	1
**Transcriptional regulator, XRE family**	1	1	1
**Tyrosine type site-specific recombinase**	14	-	15
**Valyl-tRNA synthetase**	1	1	1
**Zinc ABC transporter, periplasmic-binding protein ZnuA**	1	1	1
**Total of above**	324	42	453
**Hypothetical protein**	765	26	980
**Intergenic region**	211	20	287

## Discussion

Projects for determining the complete genomes of *P*. *gingivalis* have provided information that was difficult to obtain while using traditional research methods that largely depended on culture techniques [31–33]. The presence of CRISPR/Cas systems in *P*. *gingivalis* genomes is an example of such information, although it remains unclear why potential targets of most spacers are unknown and the predicted potential targets are predominantly in their own genomes [35]. If the immune systems in *P*. *gingivalis* are involved in its genome plasticity, then the CRISPR/Cas would be more significant than innate immune systems such as the restriction-modification system because the adaptive immune systems are able to recognize the invasion of exogenous nucleic acids. In this study, we identified novel CRISPR/Cas arrays in *Porphyromonas* genomes and previously known ones in *P*. *gingivalis* (Fig 1, S5 Table). The *cas* genes identified in this study were classified into three types or were unclassified because of the genes did not have the typical features of particular types (Fig 1, S5 Table). The types I-C and II-C observed in this study target DNA, whereas type III-B is able to target both DNA and ribonucleic acid [48]. A previous study [49] demonstrated that the known four CRISPR arrays were transcriptionally active in *P*. *gingivalis* W83, one of which utilized a nucleotide sequence NGG as a PAM. In another study [50], the CRISPR inhibition of genetic exchange was suggested for *P*. *gingivalis* during active growth phase. Based on our findings and previous research findings, the newly identified CRISPR arrays in this study may have been transcribed and used for adaptive immunity against exogenous nucleic acids.

However, the findings from our previous study [35] on *P*. *gingivalis* CRISPR, which identified very few potential targets, were inconsistent with the activity of CRISPR suggested above. The investigation in this report used 7,331 spacers in *Porphyromonas* genomes, which was approximately 3.4 times more abundant than in the aforementioned study (2,150 spacers in the *P*. *gingivalis* genomes), and we identified few potential targets (1.1% of all spacers; S6 Table) in which those in the genus *Porphyromonas* were excluded. The broadness of the bacterial species of potential targets (Table 1) may correspond to a variety of bacterial species in the human oral cavity, which was greater than 1,000 [51], although we currently do not understand whether these targeted species actually colonize in the oral cavity or just pass through by contamination into the oral cavity from the environment. On the other hand, including *Porphyromonas* genomes in the search of potential targets of CRISPR resulted in an unexpectedly large number of spacers, and accounted for 24.9% of all targets (S7 Table). These potential targets included CDSs of various functions and those encoding hypothetical proteins or those related to exogenous elements (Table 2). We observed a predominance of potential targets in the *Porphyromonas* genomes with a perfect nucleotide match and identified targets with mismatches of 1–7 nucleotides (Fig 2). These findings of nucleotide mismatches could be because of a mutation in the spacers or their potential targets [52], which are also the situations for the PAM that we could not identify in this study (S4 Fig), or because of the presence of corresponding nucleotide sequences that have not been detected previously.

**Fig 3:**
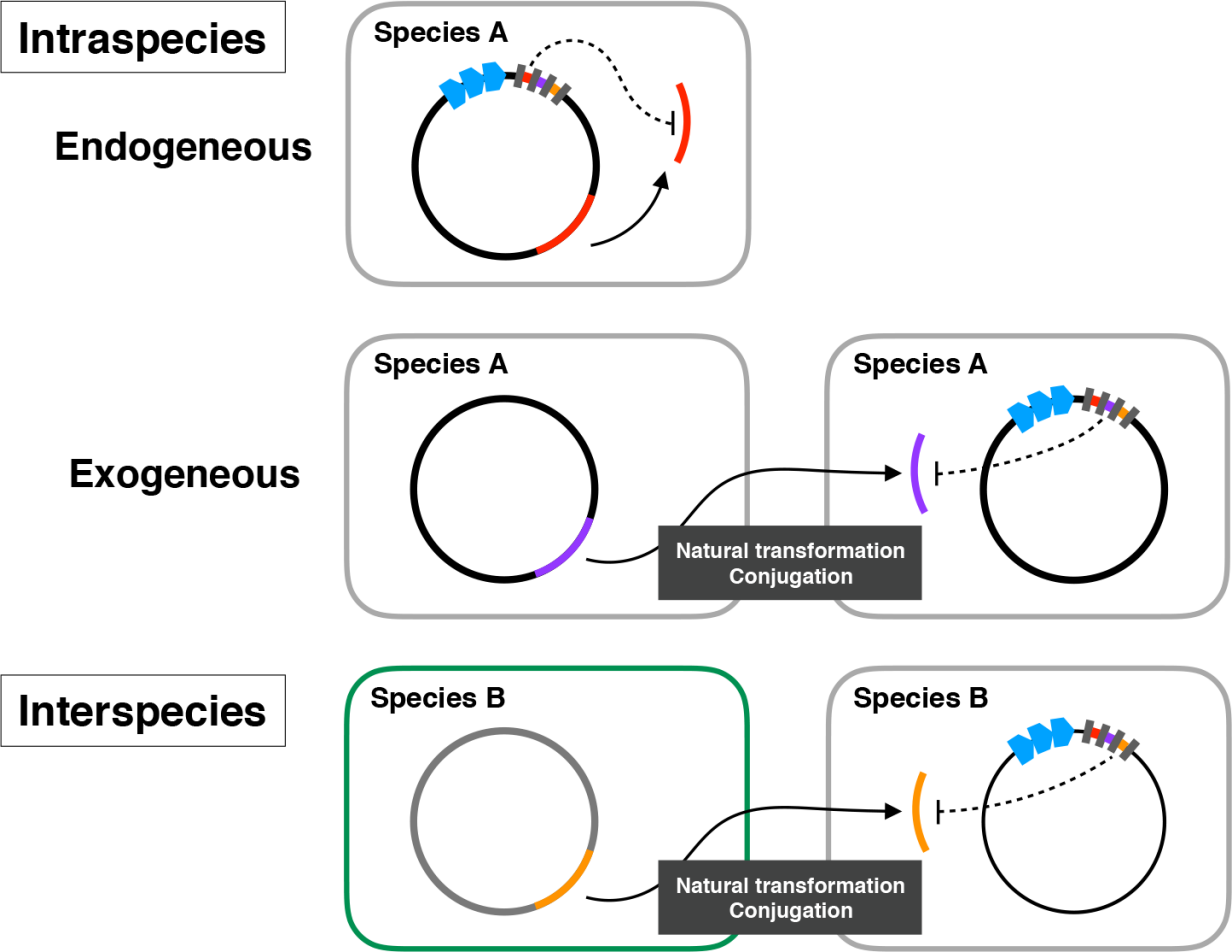
A schematic view of the locational relationships between CRISPR spacers and their potential targets. Endogenous interference of *Porphyromonas* CRISPR/Cas is supposed for the CRISPR spacer and its target, both of which are in the same species A. Exogenous interference is supposed for the spacer in the species A and its target that is introduced from another cell of the species A by the mechanisms such as natural transformation and conjugation. These endogenous and exogenous interferences are referred to as intraspecies interference in this paper. Interspecies interference is supposed for the spacer in the species A and its target introduced from the species B.

Considering the findings of the potential targets in the *Porphyromonas* genomes, we classified potential CRISPR interference into intraspecies and interspecies, and further classified the intraspecies interference into endogenous and exogenous (Fig 3). *Porphyromonas* CRISPR arrays with endogenous potential targets may be involved in the regulation of endogenous gene expression [53]. There might be another possibility that the targets have their origins in the exogenous genomes but are the parts of *Porphyromonas* genomes, which would avoid cell death via CRISPR interference. This would be possibly due to nucleotide mismatches between the spacers and their targets, changes of PAMs, or other unknown mechanisms. Besides these possibilities, the reason why metabolism-related CDSs were targeted by the CRISPR endogenously needs to be clarified by further investigations for the precise mechanisms of CRISPR/Cas in the genus *Porphyromonas*. Meanwhile, we also identified potential exogenous targets and interspecies targets (Figs 2 and 3, Table 2). This finding may be associated with the regulation of bacterial diversification suggested in a previous study [35] in which CRISPR/Cas may prevent the genome from undergoing rearrangements by transposition of the exogenous elements and homologous recombination with the exogenous DNA. The exogenous and interspecies interference would remove the exogenous DNA which has similar or the same sequences with the own genomes and is a potential source of recombination. The potential targets in the phage-related CDSs, probably in the conjugative transposons, would support the CRISPR interference for the regulation of bacterial diversification. Overall, we remarkably demonstrated a relationship between the CRISPR spacers and potential intraspecies, interspecies, endogenous, or exogenous targets (Fig 3).

Nearly all CRISPR arrays in this study were unique to the genus *Porphyromonas*, whereas the repeat of the type 32.2 CRISPR arrays was shared with *Parabacteroides distasonis* and *Paenibacillus stellifer*, which have been isolated from human feces and a food-packaging paperboard, respectively [54–56]. The presence of these two bacterial species in the human oral cavity has not described previously, and little is known about them (e.g., the oral administration of *Parabacteroides distasonis* enhances dextran sulfate sodium-induced colitis in mice) [57]. Type 32.2 CRISPR arrays may be transferred between the genus *Porphyromonas* and these two species. Horizontal transfer could be a possible mechanism that provides the same repeat type into different bacterial species with regard to the disagreement in the phylogenetic relationship between the taxonomy of bacteria and the constructed tree from CRISPR/Cas [58]. Such horizontal transfer of CRISPR arrays was reported in a wide range of bacteria and archaea [58–60]. The transfer of CRISPR arrays may also occur among *Porphyromonas* species because the same repeat types were found in different species, especially those with different hosts (S2 Table). For example, type 30.1 CRISPR arrays exist in *P*. *gingivalis* in humans [22], in *P*. *gulae* in various animals such as dogs and cats [61], and in *P*. *macacae* in monkeys and dogs [62]. These CRISPR arrays may have their origin before the diversification of host animals if the bacterial species with these CRISPR arrays diverged along with their hosts. How the CRISPR arrays are transferred among these bacterial species with different hosts is unknown; however, they may exist in hosts in which they have not been previously detected. In fact, *P*. *gingivalis* has been isolated from a hospital bathroom sink drain [63], despite its fastidious growth habits and restricted habitats in the human oral cavity [22, 64].

In addition to bacterial microbiome, the viral community is a growing concern in microbiology because of its various effects on human health by interacting with the host, other viruses, and bacteria [65, 66]. The salivary virome may be a reservoir for pathogenic gene function [67] and be targeted by CRISPR/Cas in oral bacteria as exogenous elements for the bacteria. However, little is known about virulent phages even for *Streptococcus mutans* [68], the bacterial species predominant in oral microbiota. Moreover, *S*. *mutans* CRISPR/Cas was shown to be involved in stress response and dissemination of antibiotic resistance genes [68], which is one of noncanonical function of CRISPR/Cas. Considering such findings, it is possible that the oral viromes are not significant reservoirs of potential targets of *Porphyromonas* CRISPR/Cas, whereas *Porphyromonas* genomes would indeed be the main reservoirs of potential targets. In addition, the repertoire of the phage-related CDSs with potential targets suggests the post-transcriptional regulation of exogenous phages by the *Porphyromonas* CRISPR/Cas, although these CDSs were in the *Porphyromonas* genomes. The *Porphyromonas* CRISPR/Cas would be associated with interference for the following representatives of post-transcriptional events of exogenous phages: 1) portal (the portal proteins), 2) gene transcription (the antirepressor protein), 3) replication (the replication initiation protein), 4) virion morphogenesis (the virion morphogenesis protein and the tail length tape-measure protein), and 5) genome packaging (the terminase proteins) (Table 2). The *Porphyromonas* species may have CRISPR/Cas for specific interference for various post-transcriptional events of exogenous phages. Future studies will need to address actual function of *Porphyromonas* CRISPR/Cas and its roles in maintaining genome plasticity and taxonomic relationships among *Porphyromonas* species.

## Supporting information

S1 Fig The number of CRISPR spacers per strain. The mean number of CRISPR spacers among strains is shown for (A) each *Porphyromonas* species and (B) each repeat type. Error bars indicate standard deviations among strains.
(PDF)

S2 Fig Nucleotide polymorphisms of CRISPR repeats in 18 repeat types. In the WebLogo illustration, the nucleotides are indicated by alphabetical letters in four different colors. At each nucleotide position, nucleotide conservation is indicated by bits (i.e., the height of the letter). The bit is 2.0 if a certain nucleotide is completely conserved.
(PDF)

S3 Fig Genetic organization of all CRISPR arrays and *cas* genes in 110 *Porphyromonas* genomes. The organization is the same as in Fig 1. The number in each CRISPR array indicates the number of spacers. The CRISPR arrays occupying the whole length of the contig are excluded from the illustration.
(PDF)

S4 Fig Nucleotide polymorphisms of upstream and downstream 50-bp of the potential targets in 18 repeat types. The WebLogo illustration is shown for upstream and downstream and for each repeat type. The illustration is prepared as in S1 Fig.
(PDF)

S1 Table Genetic information of *Porphyromonas* strains used in this study. (XLSX)

S2 Table The number of CRISPR loci in 23 *Porphyromonas* species. (XLSX)

S3 Table The number of CRISPR spacers in 23 *Porphyromonas* species. (XLSX)

S4 Table *Porphyromonas* CRISPR spacers identified in this study. (XLSX)

S5 Table CRISPR types found in this study. (XLSX)

S6 Table The number of *Porphyromonas* CRISPR spacers for which the targets are described in the NCBI Nucleotide Collection. (XLSX)

S7 Table The number of *Porphyromonas* CRISPR spacers with targets in the *Porphyromonas* genomes used in this study. (XLSX)

S8 Table The number of CRISPR targets in the *Porphyromonas* genomes used in this study. (XLSX)

## References

[1] Didelot X, Maiden MC. Impact of recombination on bacterial evolution. Trends Microbiol. 2010;18(7):315–22. Epub 2010/05/11. doi: 10.1016/j.tim.2010.04.002 ; PubMed Central PMCID: PMCPMC3985120. 20452218 10.1016/j.tim.2010.04.002 PMC3985120

[2] Vos M. Why do bacteria engage in homologous recombination? Trends Microbiol. 2009;17(6):226–32. Epub 2009/05/26. doi: 10.1016/j.tim.2009.03.001 . 19464181 10.1016/j.tim.2009.03.001

[3] Darmon E, Leach DR. Bacterial genome instability. Microbiol Mol Biol Rev. 2014;78(1):1–39. Epub 2014/03/07. doi: 10.1128/MMBR.00035-13 ; PubMed Central PMCID: PMCPMC3957733. 24600039 10.1128/MMBR.00035-13 PMC3957733

[4] Hanage WP, Fraser C, Spratt BG. The impact of homologous recombination on the generation of diversity in bacteria. J Theor Biol. 2006;239(2):210–9. Epub 2005/10/21. doi: 10.1016/j.jtbi.2005.08.035 . 16236325 10.1016/j.jtbi.2005.08.035

[5] Schoen C, Tettelin H, Parkhill J, Frosch M. Genome flexibility in *Neisseria meningitidis*. Vaccine. 2009;27 Suppl 2:B103–11. Epub 2009/05/30. doi: 10.1016/j.vaccine.2009.04.064 ; PubMed Central PMCID: PMCPMC3898611. 19477564 10.1016/j.vaccine.2009.04.064 PMC3898611

[6] Kong Y, Ma JH, Warren K, Tsang RS, Low DE, Jamieson FB, et al Homologous recombination drives both sequence diversity and gene content variation in Neisseria meningitidis. Genome Biol Evol. 2013;5(9):1611–27. doi: 10.1093/gbe/evt116 ; PubMed Central PMCID: PMC3787668. 23902748 10.1093/gbe/evt116 PMC3787668

[7] Baltrus DA, Blaser MJ, Guillemin K. *Helicobacter pylori* genome plasticity. Genome dynamics. 2009;6:75–90. Epub 2009/08/22. doi: 10.1159/000235764 . 19696495 10.1159/000235764

[8] Smith JM, Smith NH, O'Rourke M, Spratt BG. How clonal are bacteria? Proc Natl Acad Sci U S A. 1993;90(10):4384–8. Epub 1993/05/15. ; PubMed Central PMCID: PMCPMC46515. 8506277 10.1073/pnas.90.10.4384 PMC46515

[9] Ogier JC, Calteau A, Forst S, Goodrich-Blair H, Roche D, Rouy Z, et al Units of plasticity in bacterial genomes: new insight from the comparative genomics of two bacteria interacting with invertebrates, *Photorhabdus* and *Xenorhabdus*. BMC Genomics. 2010;11:568 Epub 2010/10/19. doi: 10.1186/1471-2164-11-568 ; PubMed Central PMCID: PMCPMC3091717. 20950463 10.1186/1471-2164-11-568 PMC3091717

[10] Salyers AA, Shoemaker NB, Stevens AM, Li LY. Conjugative transposons: an unusual and diverse set of integrated gene transfer elements. Microbiol Rev. 1995;59(4):579–90. Epub 1995/12/01. ; PubMed Central PMCID: PMCPMC239388. 8531886 10.1128/mr.59.4.579-590.1995 PMC239388

[11] Goessweiner-Mohr N, Arends K, Keller W, Grohmann E. Conjugation in Gram-positive bacteria. Microbiology spectrum. 2014;2(4):Plas-0004-2013. Epub 2015/06/25. doi: 10.1128/microbiolspec.PLAS-0004-2013 . 26104193 10.1128/microbiolspec.PLAS-0004-2013

[12] Ochman H, Lawrence JG, Groisman EA. Lateral gene transfer and the nature of bacterial innovation. Nature. 2000;405(6784):299–304. Epub 2000/06/01. doi: 10.1038/35012500 . 10830951 10.1038/35012500

[13] Johnston C, Martin B, Fichant G, Polard P, Claverys JP. Bacterial transformation: distribution, shared mechanisms and divergent control. Nat Rev Microbiol. 2014;12(3):181–96. Epub 2014/02/11. doi: 10.1038/nrmicro3199 . 24509783 10.1038/nrmicro3199

[14] Mell JC, Redfield RJ. Natural competence and the evolution of DNA uptake specificity. J Bacteriol. 2014;196(8):1471–83. Epub 2014/02/04. doi: 10.1128/JB.01293-13 ; PubMed Central PMCID: PMCPMC3993363. 24488316 10.1128/JB.01293-13 PMC3993363

[15] Sorek R, Lawrence CM, Wiedenheft B. CRISPR-mediated adaptive immune systems in bacteria and archaea. Annu Rev Biochem. 2013;82:237–66. doi: 10.1146/annurev-biochem-072911-172315 . 23495939 10.1146/annurev-biochem-072911-172315

[16] Vasu K, Nagaraja V. Diverse functions of restriction-modification systems in addition to cellular defense. Microbiol Mol Biol Rev. 2013;77(1):53–72. Epub 2013/03/09. doi: 10.1128/MMBR.00044-12 ; PubMed Central PMCID: PMCPMC3591985. 23471617 10.1128/MMBR.00044-12 PMC3591985

[17] Makarova KS, Haft DH, Barrangou R, Brouns SJ, Charpentier E, Horvath P, et al Evolution and classification of the CRISPR-Cas systems. Nat Rev Microbiol. 2011;9(6):467–77. doi: 10.1038/nrmicro2577 ; PubMed Central PMCID: PMC3380444. 21552286 10.1038/nrmicro2577 PMC3380444

[18] Sorek R, Kunin V, Hugenholtz P. CRISPR—a widespread system that provides acquired resistance against phages in bacteria and archaea. Nat Rev Microbiol. 2008;6(3):181–6. Epub 2007/12/25. doi: 10.1038/nrmicro1793 . 18157154 10.1038/nrmicro1793

[19] Jorth P, Whiteley M. An evolutionary link between natural transformation and CRISPR adaptive immunity. MBio. 2012;3(5). doi: 10.1128/mBio.00309-12 ; PubMed Central PMCID: PMC3484387. 23033473 10.1128/mBio.00309-12 PMC3484387

[20] Aklujkar M, Lovley DR. Interference with histidyl-tRNA synthetase by a CRISPR spacer sequence as a factor in the evolution of *Pelobacter carbinolicus*. BMC Evol Biol. 2010;10:230 doi: 10.1186/1471-2148-10-230 ; PubMed Central PMCID: PMC2923632. 20667132 10.1186/1471-2148-10-230 PMC2923632

[21] Cady KC, O'Toole GA. Non-identity-mediated CRISPR-bacteriophage interaction mediated via the Csy and Cas3 proteins. J Bacteriol. 2011;193(14):3433–45. doi: 10.1128/JB.01411-10 ; PubMed Central PMCID: PMC3133329. 21398535 10.1128/JB.01411-10 PMC3133329

[22] Lamont RJ, Jenkinson HF. Life below the gum line: pathogenic mechanisms of *Porphyromonas gingivalis*. Microbiol Mol Biol Rev. 1998;62(4):1244–63. Epub 1998/12/05. ; PubMed Central PMCID: PMCPMC98945. 9841671 10.1128/mmbr.62.4.1244-1263.1998 PMC98945

[23] Koehler A, Karch H, Beikler T, Flemmig TF, Suerbaum S, Schmidt H. Multilocus sequence analysis of *Porphyromonas gingivalis* indicates frequent recombination. Microbiology. 2003;149(Pt 9):2407–15. Epub 2003/09/02. doi: 10.1099/mic.0.26267-0 . 12949166 10.1099/mic.0.26267-0

[24] Enersen M. *Porphyromonas gingivalis*: a clonal pathogen?: Diversities in housekeeping genes and the major fimbriae gene. J Oral Microbiol. 2011;3 doi: 10.3402/jom.v3i0.8487 ; PubMed Central PMCID: PMC3223970. 22125739 10.3402/jom.v3i0.8487 PMC3223970

[25] Tribble GD, Lamont GJ, Progulske-Fox A, Lamont RJ. Conjugal transfer of chromosomal DNA contributes to genetic variation in the oral pathogen *Porphyromonas gingivalis*. J Bacteriol. 2007;189(17):6382–8. doi: 10.1128/JB.00460-07 ; PubMed Central PMCID: PMC1951918. 17573478 10.1128/JB.00460-07 PMC1951918

[26] Tribble GD, Rigney TW, Dao DH, Wong CT, Kerr JE, Taylor BE, et al Natural competence is a major mechanism for horizontal DNA transfer in the oral pathogen *Porphyromonas gingivalis*. MBio. 2012;3(1). doi: 10.1128/mBio.00231-11 ; PubMed Central PMCID: PMC3268665. 22294679 10.1128/mBio.00231-11 PMC3268665

[27] Naito M, Sato K, Shoji M, Yukitake H, Ogura Y, Hayashi T, et al Characterization of the *Porphyromonas gingivalis* conjugative transposon CTnPg1: determination of the integration site and the genes essential for conjugal transfer. Microbiology. 2011;157(Pt 7):2022–32. doi: 10.1099/mic.0.047803-0 . 21527470 10.1099/mic.0.047803-0

[28] Salmond GP, Fineran PC. A century of the phage: past, present and future. Nat Rev Microbiol. 2015;13(12):777–86. Epub 2015/11/10. doi: 10.1038/nrmicro3564 . 26548913 10.1038/nrmicro3564

[29] Tribble GD, Kerr JE, Wang BY. Genetic diversity in the oral pathogen *Porphyromonas gingivalis*: molecular mechanisms and biological consequences. Future Microbiol. 2013;8(5):607–20. Epub 2013/05/07. doi: 10.2217/fmb.13.30 ; PubMed Central PMCID: PMCPMC3808122. 23642116 10.2217/fmb.13.30 PMC3808122

[30] Cugini C, Klepac-Ceraj V, Rackaityte E, Riggs JE, Davey ME. *Porphyromonas gingivalis*: keeping the pathos out of the biont. J Oral Microbiol. 2013;5 doi: 10.3402/jom.v5i0.19804 ; PubMed Central PMCID: PMC3617648. 23565326 10.3402/jom.v5i0.19804 PMC3617648

[31] Nelson KE, Fleischmann RD, DeBoy RT, Paulsen IT, Fouts DE, Eisen JA, et al Complete genome sequence of the oral pathogenic bacterium *Porphyromonas gingivalis* strain W83. J Bacteriol. 2003;185(18):5591–601. doi: 10.1128/JB.185.18.5591-5601.2003 12949112 10.1128/JB.185.18.5591-5601.2003 PMC193775

[32] Naito M, Hirakawa H, Yamashita A, Ohara N, Shoji M, Yukitake H, et al Determination of the genome sequence of *Porphyromonas gingivalis* strain ATCC 33277 and genomic comparison with strain W83 revealed extensive genome rearrangements in *P*. *gingivalis*. DNA Res. 2008;15(4):215–25. doi: 10.1093/dnares/dsn013 ; PubMed Central PMCID: PMCPMC2575886. 18524787 10.1093/dnares/dsn013 PMC2575886

[33] Watanabe T, Maruyama F, Nozawa T, Aoki A, Okano S, Shibata Y, et al Complete genome sequence of the bacterium *Porphyromonas gingivalis* TDC60, which causes periodontal disease. J Bacteriol. 2011;193(16):4259–60. doi: 10.1128/JB.05269-11 ; PubMed Central PMCID: PMC3147703. 21705612 10.1128/JB.05269-11 PMC3147703

[34] Samson JE, Magadan AH, Moineau S. The CRISPR-Cas immune system and genetic transfers: reaching an equilibrium. Microbiology spectrum. 2015;3(1):Plas-0034-2014. Epub 2015/06/25. doi: 10.1128/microbiolspec.PLAS-0034-2014 . 26104549 10.1128/microbiolspec.PLAS-0034-2014

[35] Watanabe T, Nozawa T, Aikawa C, Amano A, Maruyama F, Nakagawa I. CRISPR regulation of intraspecies diversification by limiting IS transposition and intercellular recombination. Genome Biol Evol. 2013;5(6):1099–114. doi: 10.1093/gbe/evt075 ; PubMed Central PMCID: PMC3698921. 23661565 10.1093/gbe/evt075 PMC3698921

[36] Bolger AM, Lohse M, Usadel B. Trimmomatic: a flexible trimmer for Illumina sequence data. Bioinformatics. 2014;30(15):2114–20. Epub 2014/04/04. doi: 10.1093/bioinformatics/btu170 ; PubMed Central PMCID: PMCPMC4103590. 24695404 10.1093/bioinformatics/btu170 PMC4103590

[37] Aronesty E. Comparison of sequencing utility programs. The Open Bioinformatics Journal. 2013;7(1):1–8. doi: 10.2174/1875036201307010001

[38] Zerbino DR, Birney E. Velvet: algorithms for de novo short read assembly using de Bruijn graphs. Genome Res. 2008;18(5):821–9. Epub 2008/03/20. doi: 10.1101/gr.074492.107 ; PubMed Central PMCID: PMCPMC2336801. 18349386 10.1101/gr.074492.107 PMC2336801

[39] Aziz RK, Bartels D, Best AA, DeJongh M, Disz T, Edwards RA, et al The RAST Server: rapid annotations using subsystems technology. BMC Genomics. 2008;9:75 Epub 2008/02/12. doi: 10.1186/1471-2164-9-75 ; PubMed Central PMCID: PMCPMC2265698. 18261238 10.1186/1471-2164-9-75 PMC2265698

[40] Overbeek R, Olson R, Pusch GD, Olsen GJ, Davis JJ, Disz T, et al The SEED and the Rapid Annotation of microbial genomes using Subsystems Technology (RAST). Nucleic Acids Res. 2014;42(Database issue):D206–14. Epub 2013/12/03. doi: 10.1093/nar/gkt1226 ; PubMed Central PMCID: PMCPMC3965101. 24293654 10.1093/nar/gkt1226 PMC3965101

[41] Bland C, Ramsey TL, Sabree F, Lowe M, Brown K, Kyrpides NC, et al CRISPR recognition tool (CRT): a tool for automatic detection of clustered regularly interspaced palindromic repeats. BMC Bioinformatics. 2007;8:209 Epub 2007/06/20. doi: 10.1186/1471-2105-8-209 ; PubMed Central PMCID: PMCPmc1924867. 17577412 10.1186/1471-2105-8-209 PMC1924867

[42] Ohyama A, Kurokawa K, Enai K, Saitoh H, Kanaya S, Altaf-Ul-Amin M, et al Bioinformatics tool for genomic era: a step towards the *in silico* experiments—focused on molecular cloning. J Comp Aid Chem 2006;7:102–15.

[43] Li W, Godzik A. Cd-hit: a fast program for clustering and comparing large sets of protein or nucleotide sequences. Bioinformatics. 2006;22(13):1658–9. doi: 10.1093/bioinformatics/btl158 . 16731699 10.1093/bioinformatics/btl158

[44] Fu L, Niu B, Zhu Z, Wu S, Li W. CD-HIT: accelerated for clustering the next-generation sequencing data. Bioinformatics. 2012;28(23):3150–2. Epub 2012/10/13. doi: 10.1093/bioinformatics/bts565 ; PubMed Central PMCID: PMCPmc3516142. 23060610 10.1093/bioinformatics/bts565 PMC3516142

[45] Lange SJ, Alkhnbashi OS, Rose D, Will S, Backofen R. CRISPRmap: an automated classification of repeat conservation in prokaryotic adaptive immune systems. Nucleic Acids Res. 2013;41(17):8034–44. Epub 2013/07/19. doi: 10.1093/nar/gkt606 ; PubMed Central PMCID: PMCPmc3783184. 23863837 10.1093/nar/gkt606 PMC3783184

[46] Alkhnbashi OS, Costa F, Shah SA, Garrett RA, Saunders SJ, Backofen R. CRISPRstrand: predicting repeat orientations to determine the crRNA-encoding strand at CRISPR loci. Bioinformatics. 2014;30(17):i489–96. Epub 2014/08/28. doi: 10.1093/bioinformatics/btu459 ; PubMed Central PMCID: PMCPmc4147912. 25161238 10.1093/bioinformatics/btu459 PMC4147912

[47] Crooks GE, Hon G, Chandonia JM, Brenner SE. WebLogo: a sequence logo generator. Genome Res. 2004;14(6):1188–90. doi: 10.1101/gr.849004 ; PubMed Central PMCID: PMC419797. 15173120 10.1101/gr.849004 PMC419797

[48] Makarova KS, Wolf YI, Alkhnbashi OS, Costa F, Shah SA, Saunders SJ, et al An updated evolutionary classification of CRISPR-Cas systems. Nat Rev Microbiol. 2015;13(11):722–36. Epub 2015/09/29. doi: 10.1038/nrmicro3569 . 26411297 10.1038/nrmicro3569 PMC5426118

[49] Burmistrz M, Dudek B, Staniec D, Rodriguez Martinez JI, Bochtler M, Potempa J, et al Functional analysis of *Porphyromonas gingivalis* W83 CRISPR-Cas systems. J Bacteriol. 2015;197(16):2631–41. Epub 2015/05/28. doi: 10.1128/JB.00261-15 ; PubMed Central PMCID: PMCPmc4507336. 26013482 10.1128/JB.00261-15 PMC4507336

[50] Phillips P, Progulske-Fox A, Grieshaber S, Grieshaber N. Expression of *Porphyromonas gingivalis* small RNA in response to hemin availability identified using microarray and RNA-seq analysis. FEMS Microbiol Lett. 2014;351(2):202–8. Epub 2013/11/20. doi: 10.1111/1574-6968.12320 ; PubMed Central PMCID: PMCPMC4009720. 24245974 10.1111/1574-6968.12320 PMC4009720

[51] Dewhirst FE, Chen T, Izard J, Paster BJ, Tanner AC, Yu WH, et al The human oral microbiome. J Bacteriol. 2010;192(19):5002–17. doi: 10.1128/JB.00542-10 ; PubMed Central PMCID: PMC2944498. 20656903 10.1128/JB.00542-10 PMC2944498

[52] Bhaya D, Davison M, Barrangou R. CRISPR-Cas systems in bacteria and archaea: versatile small RNAs for adaptive defense and regulation. Annu Rev Genet. 2011;45:273–97. doi: 10.1146/annurev-genet-110410-132430 . 22060043 10.1146/annurev-genet-110410-132430

[53] Stern A, Keren L, Wurtzel O, Amitai G, Sorek R. Self-targeting by CRISPR: gene regulation or autoimmunity? Trends Genet. 2010;26(8):335–40. doi: 10.1016/j.tig.2010.05.008 ; PubMed Central PMCID: PMC2910793. 20598393 10.1016/j.tig.2010.05.008 PMC2910793

[54] Sakamoto M, Benno Y. Reclassification of *Bacteroides distasonis*, *Bacteroides goldsteinii* and *Bacteroides merdae* as *Parabacteroides distasonis* gen. nov., comb. nov., *Parabacteroides goldsteinii* comb. nov. and *Parabacteroides merdae* comb. nov. Int J Syst Evol Microbiol. 2006;56(Pt 7):1599–605. Epub 2006/07/11. doi: 10.1099/ijs.0.64192-0 . 16825636 10.1099/ijs.0.64192-0

[55] Eggerth AH, Gagnon BH. The *Bacteroides* of human feces. J Bacteriol. 1933;25(4):389–413. Epub 1933/04/01. ; PubMed Central PMCID: PMCPMC533498. 16559622 10.1128/jb.25.4.389-413.1933 PMC533498

[56] Suominen I, Sproer C, Kampfer P, Rainey FA, Lounatmaa K, Salkinoja-Salonen M. *Paenibacillus stellifer* sp. nov., a cyclodextrin-producing species isolated from paperboard. Int J Syst Evol Microbiol. 2003;53(Pt 5):1369–74. Epub 2003/09/18. doi: 10.1099/ijs.0.02277-0 . 13130020 10.1099/ijs.0.02277-0

[57] Dziarski R, Park SY, Kashyap DR, Dowd SE, Gupta D. Pglyrp-regulated gut microflora *Prevotella falsenii*, *Parabacteroides distasonis* and *Bacteroides eggerthii* enhance and *Alistipes finegoldii* attenuates colitis in mice. PLoS One. 2016;11(1):e0146162 Epub 2016/01/05. doi: 10.1371/journal.pone.0146162 ; PubMed Central PMCID: PMCPMC4699708. 26727498 10.1371/journal.pone.0146162 PMC4699708

[58] Karginov FV, Hannon GJ. The CRISPR system: small RNA-guided defense in bacteria and archaea. Mol Cell. 2010;37(1):7–19. doi: 10.1016/j.molcel.2009.12.033 ; PubMed Central PMCID: PMC2819186. 20129051 10.1016/j.molcel.2009.12.033 PMC2819186

[59] Chakraborty S, Snijders AP, Chakravorty R, Ahmed M, Tarek AM, Hossain MA. Comparative network clustering of direct repeats (DRs) and *cas* genes confirms the possibility of the horizontal transfer of CRISPR locus among bacteria. Mol Phylogenet Evol. 2010;56(3):878–87. Epub 2010/06/29. doi: 10.1016/j.ympev.2010.05.020 . 20580935 10.1016/j.ympev.2010.05.020

[60] Godde JS, Bickerton A. The repetitive DNA elements called CRISPRs and their associated genes: evidence of horizontal transfer among prokaryotes. J Mol Evol. 2006;62(6):718–29. Epub 2006/04/14. doi: 10.1007/s00239-005-0223-z . 16612537 10.1007/s00239-005-0223-z

[61] Fournier D, Mouton C, Lapierre P, Kato T, Okuda K, Menard C. *Porphyromonas gulae* sp. nov., an anaerobic, gram-negative coccobacillus from the gingival sulcus of various animal hosts. Int J Syst Evol Microbiol. 2001;51(Pt 3):1179–89. Epub 2001/06/20. doi: 10.1099/00207713-51-3-1179 . 11411686 10.1099/00207713-51-3-1179

[62] Coykendall AL, Kaczmarek FS, Slots J. Genetic heterogeneity in *Bacteroides asaccharolyticus* (Holdeman and Moore 1970) Finegold and Barnes 1977 (Approved Lists, 1980) and proposal of *Bacteroides gingivalis* sp. nov. and *Bacteroides macacae* (Slots and Genco) comb. nov. Int J Syst Evol Microbiol. 1980;30(3):559–64. doi: 10.1099/00207713-30-3-559

[63] McLean JS, Lombardo MJ, Ziegler MG, Novotny M, Yee-Greenbaum J, Badger JH, et al Genome of the pathogen *Porphyromonas gingivalis* recovered from a biofilm in a hospital sink using a high-throughput single-cell genomics platform. Genome Res. 2013;23(5):867–77. doi: 10.1101/gr.150433.112 ; PubMed Central PMCID: PMC3638142. 23564253 10.1101/gr.150433.112 PMC3638142

[64] Wyss C. Growth of *Porphyromonas gingivalis*, *Treponema denticola*, *T*. *pectinovorum*, *T*. *socranskii*, and *T*. *vincentii* in a chemically defined medium. J Clin Microbiol. 1992;30(9):2225–9. Epub 1992/09/01. ; PubMed Central PMCID: PMCPMC265483. 1400984 10.1128/jcm.30.9.2225-2229.1992 PMC265483

[65] Zou S, Caler L, Colombini-Hatch S, Glynn S, Srinivas P. Research on the human virome: where are we and what is next. Microbiome. 2016;4(1):32 Epub 2016/06/28. doi: 10.1186/s40168-016-0177-y ; PubMed Central PMCID: PMCPMC4919837. 27341799 10.1186/s40168-016-0177-y PMC4919837

[66] Foxman EF, Iwasaki A. Genome-virome interactions: examining the role of common viral infections in complex disease. Nat Rev Microbiol. 2011;9(4):254–64. Epub 2011/03/17. doi: 10.1038/nrmicro2541 ; PubMed Central PMCID: PMCPMC3678363. 21407242 10.1038/nrmicro2541 PMC3678363

[67] Pride DT, Salzman J, Haynes M, Rohwer F, Davis-Long C, White RA 3rd, et al Evidence of a robust resident bacteriophage population revealed through analysis of the human salivary virome. Isme j. 2012;6(5):915–26. Epub 2011/12/14. doi: 10.1038/ismej.2011.169 ; PubMed Central PMCID: PMCPmc3329113. 22158393 10.1038/ismej.2011.169 PMC3329113

[68] Serbanescu MA, Cordova M, Krastel K, Flick R, Beloglazova N, Latos A, et al Role of the *Streptococcus mutans* CRISPR-Cas systems in immunity and cell physiology. J Bacteriol. 2015;197(4):749–61. Epub 2014/12/10. doi: 10.1128/JB.02333-14 ; PubMed Central PMCID: PMCPMC4334182. 25488301 10.1128/JB.02333-14 PMC4334182

