# Supplementary figures and images for: Investigation of potential targets of Porphyromonas CRISPRs among the genomes of Porphyromonas species
Source: PLoS One. 2017 Aug 24;12(8):e0183752. doi: 10.1371/journal.pone.0183752 (PMC5570325; doi:10.1371/journal.pone.0183752)

# S1 Fig

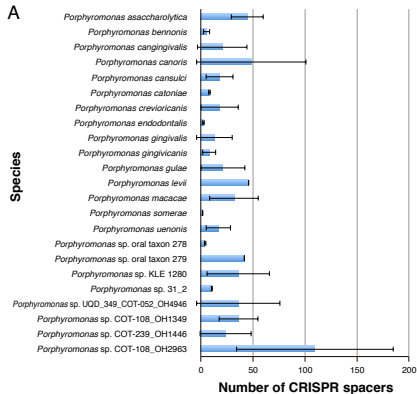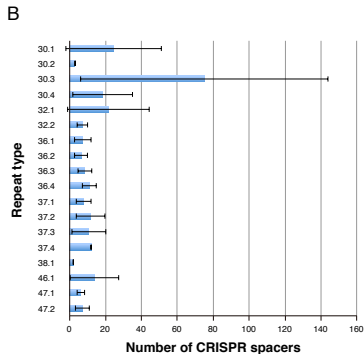

Supplement: S1 Fig — The mean number of CRISPR spacers among strains is shown for (A) each Porphyromonas species and (B) each repeat type. Error bars indicate standard deviations among strains. (PDF) [file pone.0183752.s001.pdf]

# S2 Fig

30.1

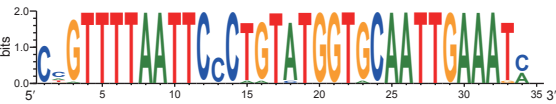

36.4

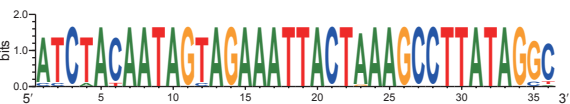

30.2

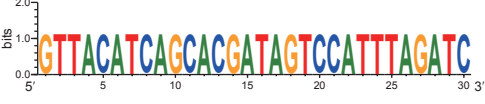

37.1

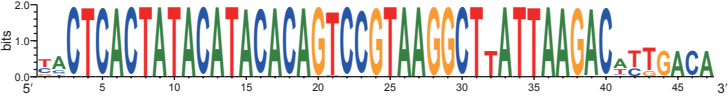

30.3

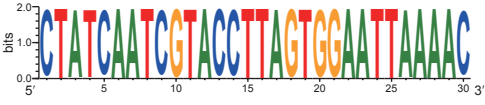

37.2

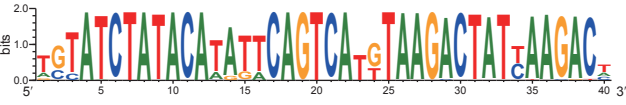

30.4

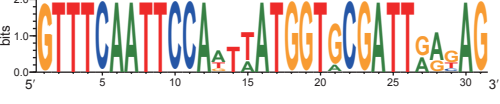

37.3

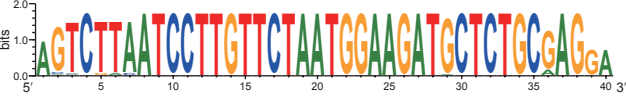

32.1

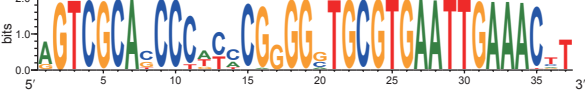

37.4

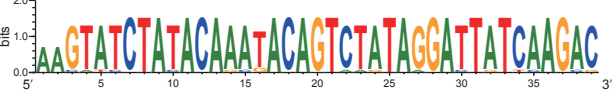

32.2

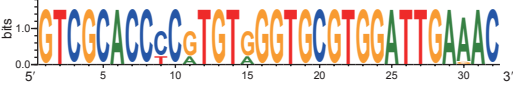

38.1

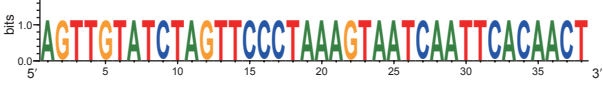

36.1

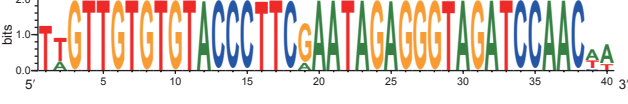

46.1

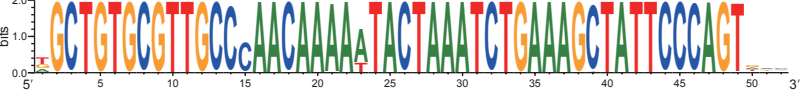

36.2

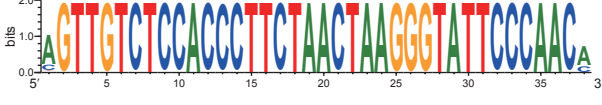

47.1

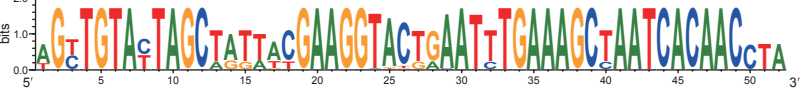

36.3

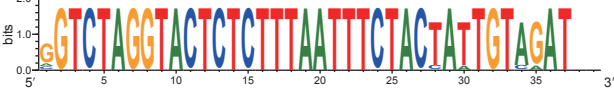

47.2

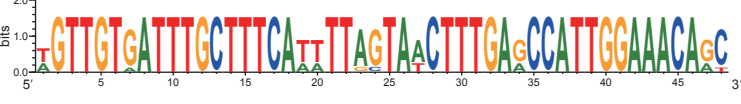

Supplement: S2 Fig — In the WebLogo illustration, the nucleotides are indicated by alphabetical letters in four different colors. At each nucleotide position, nucleotide conservation is indicated by bits (i.e., the height of the letter). The bit is 2.0 if a certain nucleotide is completely conserved. (PDF) [file pone.0183752.s002.pdf]

## S3 Fig

### 30.1

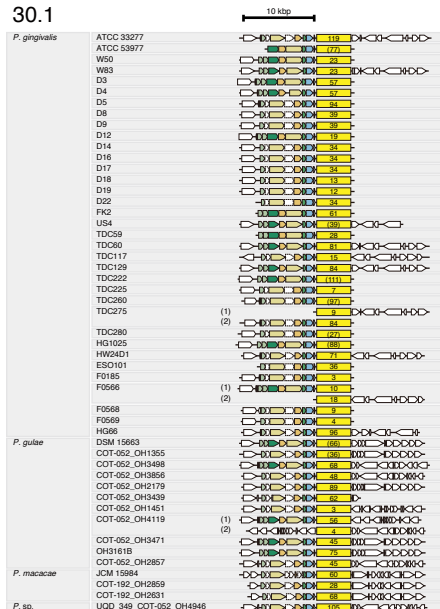

## 36.1

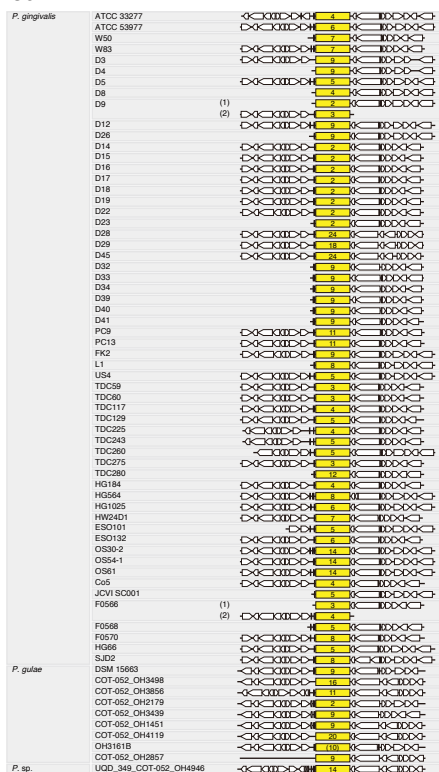

## 37.1

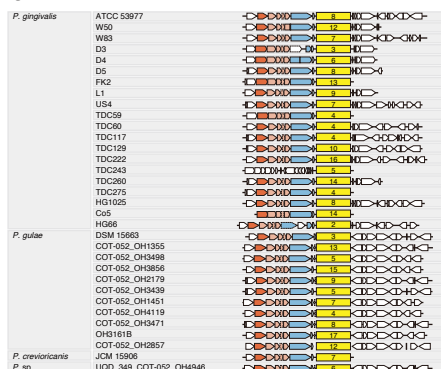

## 37.2

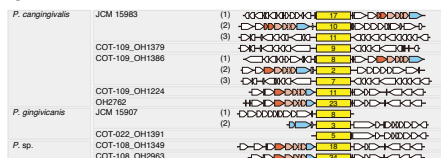

## 30.2

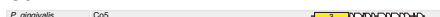

### 30.3

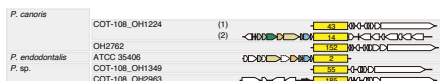

## 30.4

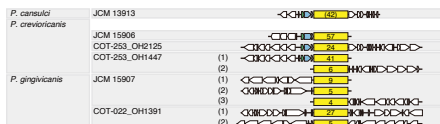

## 32.1

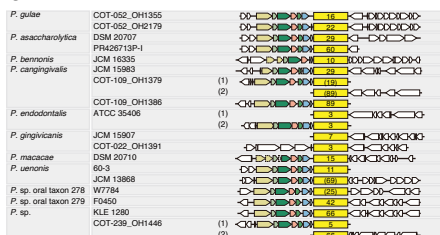

## 32.2

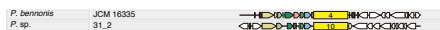

## 36.4

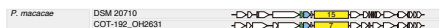

## 47.1

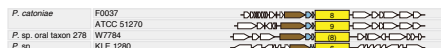

## 47.2

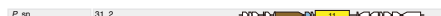

Supplement: S3 Fig — The organization is the same as in Fig 1. The number in each CRISPR array indicates the number of spacers. The CRISPR arrays occupying the whole length of the contig are excluded from the illustration. (PDF) [file pone.0183752.s003.pdf]

S4 Fig

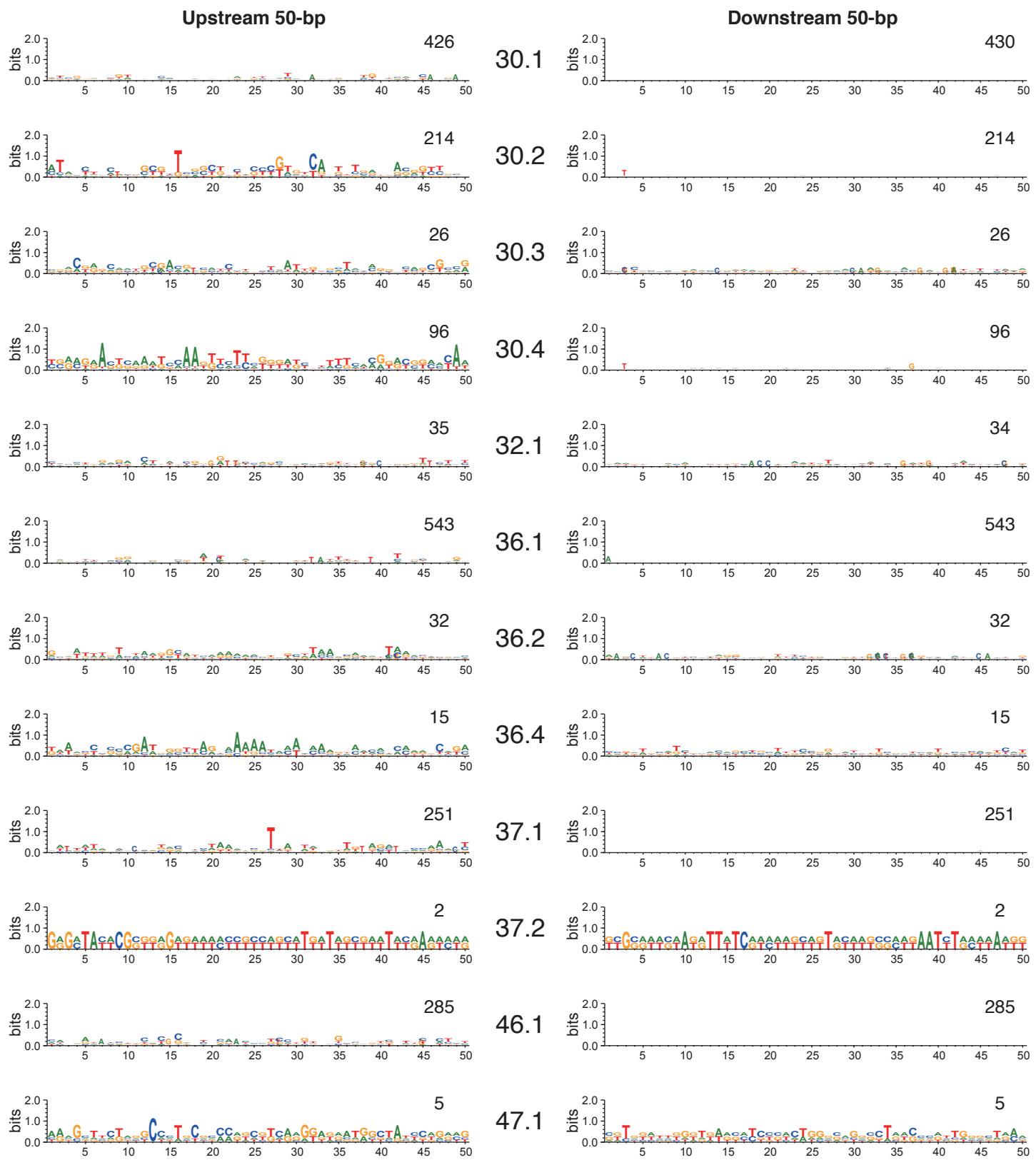

Supplement: S4 Fig — The WebLogo illustration is shown for upstream and downstream and for each repeat type. The illustration is prepared as in S1 Fig. (PDF) [file pone.0183752.s004.pdf]
